# 17-(5-Ethyl-6-methyl­heptan-2-yl)-10,13-dimethyl-2,3,4,7,8,9,10,11,12,13,14,15,16,17-tetra­deca­hydro-1*H*-cyclo­penta[*a*]phenanthrene-3,7-diol from *Chisocheton tomentosus* (*Meliaceae*)

**DOI:** 10.1107/S1600536808033163

**Published:** 2008-10-22

**Authors:** Ibrahim A. Najmuldeen, Abdul Hamid Abdul Hadi, Khalijah Awang, Khalit Mohamad, Seik Weng Ng

**Affiliations:** a Department of Chemistry, University of Malaya, 50603 Kuala Lumpur, Malaysia

## Abstract

The asymmetric unit of the title compound, C_29_H_50_O_2_, contains two mol­ecules; one mol­ecule is linked to the other by two O—H⋯O hydrogen bonds, whereas only one of the hydr­oxy groups of the second mol­ecule is involved in hydrogen bonding. This gives rise to a chain that runs along the *a* axis of the monoclinic unit cell.

## Related literature

This study is the first on *Chisocheton tomentosus*. For literature on other *Chisocheton* species, see: Awang *et al.* (2007 ▶); Bordoloi *et al.* (1993 ▶); Gunning *et al.* (1994 ▶); Inada *et al.* (1993 ▶); Phongmaykin *et al.* (2008 ▶); Tzouros *et al.* (2004 ▶).
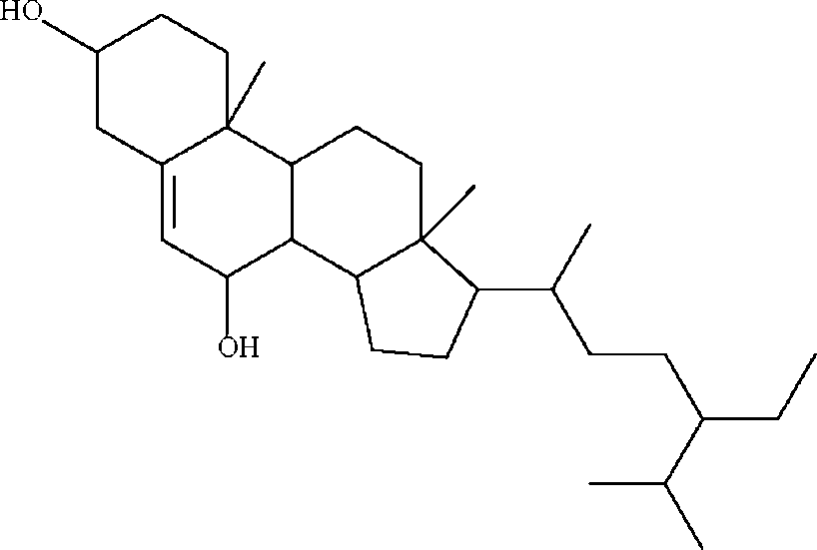


## Experimental

### 

#### Crystal data

C_29_H_50_O_2_
*M* *_r_* = 430.69
Monoclinic, 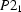

*a* = 8.1690 (1) Å
*b* = 12.2369 (2) Å
*c* = 25.6945 (4) Å
β = 93.652 (1)°
*V* = 2563.29 (7) Å^3^
*Z* = 4
Mo *K*α radiation
μ = 0.07 mm^−1^
*T* = 100 (2) K
0.45 × 0.15 × 0.03 mm

#### Data collection

Bruker SMART APEX diffractometer
Absorption correction: none
21218 measured reflections
6154 independent reflections
5389 reflections with *I* > 2σ(*I*)
*R*_int_ = 0.039

#### Refinement

*R*[*F* ^2^ > 2σ(*F* ^2^)] = 0.060
*wR*(*F* ^2^) = 0.172
*S* = 1.11
6154 reflections
575 parameters
1 restraint
H-atom parameters constrained
Δρ_max_ = 0.46 e Å^−3^
Δρ_min_ = −0.27 e Å^−3^

### 

Data collection: *APEX2* (Bruker, 2007 ▶); cell refinement: *SAINT* (Bruker, 2007 ▶); data reduction: *SAINT*; program(s) used to solve structure: *SHELXS97* (Sheldrick, 2008 ▶); program(s) used to refine structure: *SHELXL97* (Sheldrick, 2008 ▶); molecular graphics: *X-SEED* (Barbour, 2001 ▶); software used to prepare material for publication: *publCIF* (Westrip, 2008 ▶).

## Supplementary Material

Crystal structure: contains datablocks I, global. DOI: 10.1107/S1600536808033163/bt2810sup1.cif

Structure factors: contains datablocks I. DOI: 10.1107/S1600536808033163/bt2810Isup2.hkl

Additional supplementary materials:  crystallographic information; 3D view; checkCIF report

## Figures and Tables

**Table 1:** Hydrogen-bond geometry (Å, °)

*D*—H⋯*A*	*D*—H	H⋯*A*	*D*⋯*A*	*D*—H⋯*A*
O1—H1O⋯O4	0.84	1.92	2.762 (3)	178
O2—H2O⋯O3	0.84	1.87	2.686 (3)	165
O3—H3O⋯O1^i^	0.84	1.86	2.694 (3)	170

Symmetry code: (i) 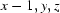.

**Fig. 1.**
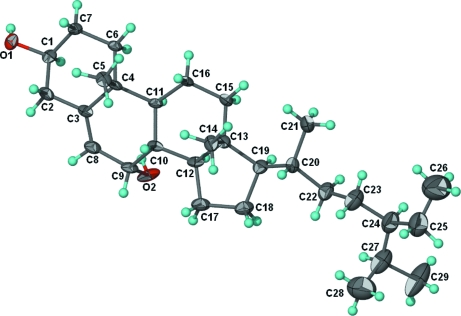
70% Probability displacement ellipsoid plot (Barbour, 2001) of C29H50O2. Hydrogen atoms are drawn as spheres of arbitrary radii.

## supplementary crystallographic information

### Experimental

Dried ground bark of *Chisocheton tomentosus* (3.5 kg) was defatted with hexane for 5 days. The solvent was removed and the plant material dried before being extracted with dichloromethane for another 5 days. The dichloromethane was removed by evaporation to give a crude material (10 g) that was subjected to column chromatography over silica gel. Separation was effected with hexane/dichloromethane (95:5 *v*/*v*). The polarity was increased with acetone, and finally with acetone/methanol to give 24 fractions. One of the later fractions was subjected to column chromatography (25:25:50 *v*/*v* acetone/dichloromethane/hexane) to give five other fractions. The third fraction yielded colorless crystals when the solvents were allowed to evaporate.

### Refinement

In the absence of significant anomalous dispersion effects, Friedel pairs were averaged. Carbon- and oxygen-bound H-atoms were placed in calculated positions (C—H 0.95–1.00, O–H 0.84 Å) and were included in the refinement in the riding model approximation, with *U*(H) set to 1.2–1.5*U*_eq_(C,O).

### Crystal data

**Table:**

C_29_H_50_O_2_	*F*(000) = 960
*M**_r_* = 430.69	*D*_x_ = 1.116 Mg m^−^^3^
Monoclinic, *P*2_1_	Mo *K*α radiation, λ = 0.71073 Å
Hall symbol: P 2yb	Cell parameters from 7107 reflections
*a* = 8.1690 (1) Å	θ = 2.3–28.3°
*b* = 12.2369 (2) Å	µ = 0.07 mm^−^^1^
*c* = 25.6945 (4) Å	*T* = 100 K
β = 93.652 (1)°	Prism, colourless
*V* = 2563.29 (7) Å^3^	0.45 × 0.15 × 0.03 mm
*Z* = 4	

### Data collection

**Table:**

Bruker SMART APEX diffractometer	5389 reflections with *I* > 2σ(*I*)
Radiation source: fine-focus sealed tube	*R*_int_ = 0.039
graphite	θ_max_ = 27.5°, θ_min_ = 0.8°
ω scans	*h* = −10→10
21218 measured reflections	*k* = −14→15
6154 independent reflections	*l* = −33→33

### Refinement

**Table:**

Refinement on *F*^2^	Primary atom site location: structure-invariant direct methods
Least-squares matrix: full	Secondary atom site location: difference Fourier map
*R*[*F*^2^ > 2σ(*F*^2^)] = 0.060	Hydrogen site location: inferred from neighbouring sites
*wR*(*F*^2^) = 0.172	H-atom parameters constrained
*S* = 1.11	*w* = 1/[σ^2^(*F*_o_^2^) + (0.1065*P*)^2^ + 0.6901*P*] where *P* = (*F*_o_^2^ + 2*F*_c_^2^)/3
6154 reflections	(Δ/σ)_max_ = 0.001
575 parameters	Δρ_max_ = 0.46 e Å^−^^3^
1 restraint	Δρ_min_ = −0.27 e Å^−^^3^

### Fractional atomic coordinates and isotropic or equivalent isotropic displacement parameters (Å^2^)

**Table:**

	*x*	*y*	*z*	*U*_iso_*/*U*_eq_	
O1	0.6382 (2)	0.7500 (2)	0.97759 (9)	0.0231 (5)	
H1O	0.6162	0.7329	0.9463	0.035*	
O2	−0.0206 (3)	0.7449 (2)	1.12076 (9)	0.0297 (6)	
H2O	−0.0118	0.7338	1.0888	0.045*	
O3	−0.0524 (3)	0.7160 (2)	1.01700 (9)	0.0293 (6)	
H3O	−0.1437	0.7326	1.0022	0.044*	
O4	0.5741 (3)	0.6943 (2)	0.87412 (9)	0.0243 (5)	
H4O	0.6488	0.6784	0.8544	0.036*	
C1	0.5203 (3)	0.7010 (3)	1.00956 (12)	0.0204 (6)	
H1	0.4114	0.6996	0.9895	0.024*	
C2	0.5068 (4)	0.7730 (3)	1.05748 (12)	0.0222 (6)	
H2A	0.6158	0.7791	1.0764	0.027*	
H2B	0.4716	0.8472	1.0463	0.027*	
C3	0.3855 (4)	0.7269 (2)	1.09368 (11)	0.0188 (6)	
C4	0.4110 (3)	0.6076 (2)	1.11020 (12)	0.0171 (6)	
C5	0.5651 (4)	0.6019 (3)	1.14821 (12)	0.0227 (6)	
H5A	0.5406	0.6343	1.1817	0.034*	
H5B	0.6549	0.6424	1.1335	0.034*	
H5C	0.5975	0.5254	1.1535	0.034*	
C6	0.4381 (4)	0.5384 (3)	1.06090 (12)	0.0197 (6)	
H6A	0.4737	0.4641	1.0720	0.024*	
H6B	0.3321	0.5311	1.0403	0.024*	
C7	0.5650 (4)	0.5859 (3)	1.02586 (13)	0.0218 (6)	
H7A	0.6743	0.5858	1.0449	0.026*	
H7B	0.5712	0.5396	0.9945	0.026*	
C8	0.2635 (4)	0.7893 (3)	1.10964 (12)	0.0230 (6)	
H8	0.2549	0.8617	1.0965	0.028*	
C9	0.1395 (4)	0.7532 (3)	1.14665 (12)	0.0214 (6)	
H9	0.1342	0.8112	1.1739	0.026*	
C10	0.1912 (4)	0.6469 (2)	1.17448 (11)	0.0178 (6)	
H10	0.2823	0.6637	1.2011	0.021*	
C11	0.2546 (4)	0.5652 (2)	1.13538 (12)	0.0175 (6)	
H11	0.1672	0.5589	1.1065	0.021*	
C12	0.0496 (4)	0.5984 (3)	1.20216 (12)	0.0197 (6)	
H12	−0.0382	0.5817	1.1744	0.024*	
C13	0.0879 (4)	0.4906 (3)	1.23062 (12)	0.0193 (6)	
C14	0.2300 (4)	0.5024 (3)	1.27277 (13)	0.0235 (6)	
H14A	0.1941	0.5472	1.3015	0.035*	
H14B	0.3237	0.5375	1.2576	0.035*	
H14C	0.2625	0.4299	1.2860	0.035*	
C15	0.1318 (4)	0.4089 (3)	1.18813 (13)	0.0234 (7)	
H15A	0.1595	0.3374	1.2044	0.028*	
H15B	0.0357	0.3985	1.1631	0.028*	
C16	0.2773 (4)	0.4499 (2)	1.15891 (13)	0.0228 (7)	
H16A	0.2971	0.3979	1.1305	0.027*	
H16B	0.3763	0.4500	1.1832	0.027*	
C17	−0.0307 (4)	0.6698 (3)	1.24235 (13)	0.0272 (7)	
H17A	−0.1021	0.7262	1.2251	0.033*	
H17B	0.0529	0.7059	1.2660	0.033*	
C18	−0.1323 (4)	0.5869 (3)	1.27215 (13)	0.0278 (7)	
H18A	−0.2507	0.5968	1.2627	0.033*	
H18B	−0.1131	0.5979	1.3102	0.033*	
C19	−0.0770 (4)	0.4705 (3)	1.25683 (13)	0.0229 (6)	
H19	−0.1584	0.4430	1.2291	0.028*	
C20	−0.0783 (4)	0.3906 (3)	1.30303 (14)	0.0249 (7)	
H20	−0.0044	0.4211	1.3320	0.030*	
C21	−0.0136 (5)	0.2765 (3)	1.28981 (17)	0.0370 (9)	
H21A	−0.0335	0.2259	1.3183	0.056*	
H21B	0.1044	0.2807	1.2852	0.056*	
H21C	−0.0706	0.2503	1.2575	0.056*	
C22	−0.2509 (4)	0.3809 (3)	1.32302 (14)	0.0310 (8)	
H22A	−0.2994	0.4549	1.3243	0.037*	
H22B	−0.3200	0.3374	1.2976	0.037*	
C23	−0.2574 (5)	0.3286 (4)	1.37654 (16)	0.0406 (9)	
H23A	−0.1983	0.3769	1.4023	0.049*	
H23B	−0.1966	0.2586	1.3763	0.049*	
C24	−0.4290 (5)	0.3056 (4)	1.39556 (15)	0.0391 (9)	
H24	−0.4861	0.2570	1.3689	0.047*	
C25	−0.4156 (6)	0.2408 (4)	1.44664 (18)	0.0487 (11)	
H25A	−0.3376	0.2787	1.4716	0.058*	
H25B	−0.5241	0.2406	1.4618	0.058*	
C26	−0.3607 (10)	0.1263 (6)	1.4410 (3)	0.081 (2)	
H26A	−0.3655	0.0884	1.4745	0.122*	
H26B	−0.2477	0.1254	1.4303	0.122*	
H26C	−0.4326	0.0892	1.4146	0.122*	
C27	−0.5338 (5)	0.4085 (5)	1.39847 (17)	0.0471 (11)	
H27	−0.5363	0.4438	1.3633	0.057*	
C28	−0.4660 (8)	0.4907 (6)	1.4369 (3)	0.0713 (16)	
H28A	−0.5273	0.5593	1.4323	0.107*	
H28B	−0.3501	0.5038	1.4312	0.107*	
H28C	−0.4760	0.4631	1.4723	0.107*	
C29	−0.7116 (6)	0.3790 (7)	1.4087 (2)	0.076 (2)	
H29A	−0.7820	0.4430	1.4020	0.114*	
H29B	−0.7173	0.3561	1.4451	0.114*	
H29C	−0.7491	0.3192	1.3855	0.114*	
C30	0.0493 (4)	0.6698 (3)	0.97992 (12)	0.0235 (7)	
H30	0.1616	0.6629	0.9975	0.028*	
C31	0.0640 (4)	0.7452 (3)	0.93332 (12)	0.0224 (6)	
H31A	−0.0460	0.7565	0.9157	0.027*	
H31B	0.1060	0.8171	0.9457	0.027*	
C32	0.1777 (3)	0.6986 (3)	0.89473 (11)	0.0181 (6)	
C33	0.1431 (3)	0.5817 (3)	0.87644 (11)	0.0160 (6)	
C34	−0.0179 (4)	0.5826 (3)	0.84128 (12)	0.0222 (6)	
H34A	−0.0994	0.6283	0.8573	0.033*	
H34B	−0.0600	0.5079	0.8373	0.033*	
H34C	0.0036	0.6123	0.8070	0.033*	
C35	0.1201 (4)	0.5101 (3)	0.92521 (12)	0.0209 (6)	
H35A	0.0827	0.4366	0.9136	0.025*	
H35B	0.2278	0.5015	0.9447	0.025*	
C36	−0.0024 (4)	0.5562 (3)	0.96208 (13)	0.0255 (7)	
H36A	−0.1129	0.5592	0.9440	0.031*	
H36B	−0.0078	0.5076	0.9927	0.031*	
C37	0.3035 (4)	0.7581 (3)	0.87996 (12)	0.0199 (6)	
H37	0.3174	0.8295	0.8941	0.024*	
C38	0.4237 (3)	0.7196 (3)	0.84267 (12)	0.0185 (6)	
H38	0.4461	0.7803	0.8181	0.022*	
C39	0.3607 (3)	0.6209 (2)	0.81143 (11)	0.0153 (5)	
H39	0.2713	0.6461	0.7859	0.018*	
C40	0.2885 (3)	0.5349 (2)	0.84685 (11)	0.0152 (6)	
H40	0.3771	0.5162	0.8740	0.018*	
C41	0.4956 (3)	0.5704 (3)	0.78089 (11)	0.0175 (6)	
H41	0.5798	0.5418	0.8074	0.021*	
C42	0.4398 (4)	0.4716 (3)	0.74682 (11)	0.0178 (6)	
C43	0.3011 (4)	0.5023 (3)	0.70591 (12)	0.0232 (6)	
H43A	0.3412	0.5572	0.6820	0.035*	
H43B	0.2081	0.5322	0.7235	0.035*	
H43C	0.2660	0.4370	0.6862	0.035*	
C44	0.3826 (4)	0.3852 (3)	0.78485 (12)	0.0206 (6)	
H44A	0.4767	0.3623	0.8085	0.025*	
H44B	0.3426	0.3201	0.7650	0.025*	
C45	0.2451 (4)	0.4284 (2)	0.81739 (12)	0.0198 (6)	
H45A	0.2180	0.3718	0.8429	0.024*	
H45B	0.1459	0.4410	0.7940	0.024*	
C46	0.5878 (4)	0.6436 (3)	0.74431 (13)	0.0255 (7)	
H46A	0.6695	0.6897	0.7641	0.031*	
H46B	0.5110	0.6914	0.7234	0.031*	
C47	0.6719 (4)	0.5619 (3)	0.70931 (13)	0.0250 (7)	
H47A	0.7920	0.5621	0.7176	0.030*	
H47B	0.6508	0.5820	0.6722	0.030*	
C48	0.6003 (4)	0.4470 (3)	0.71962 (12)	0.0205 (6)	
H48	0.6773	0.4097	0.7457	0.025*	
C49	0.5895 (4)	0.3761 (3)	0.66984 (12)	0.0239 (7)	
H49	0.5160	0.4145	0.6432	0.029*	
C50	0.5169 (5)	0.2628 (3)	0.67837 (15)	0.0329 (8)	
H50A	0.5304	0.2174	0.6475	0.049*	
H50B	0.4000	0.2698	0.6842	0.049*	
H50C	0.5739	0.2286	0.7089	0.049*	
C51	0.7592 (4)	0.3659 (3)	0.64791 (13)	0.0287 (7)	
H51A	0.8212	0.4342	0.6555	0.034*	
H51B	0.8197	0.3055	0.6661	0.034*	
C52	0.7534 (5)	0.3448 (4)	0.58952 (15)	0.0406 (10)	
H52A	0.6766	0.3977	0.5720	0.049*	
H52B	0.7084	0.2707	0.5827	0.049*	
C53	0.9195 (5)	0.3535 (4)	0.56493 (15)	0.0391 (9)	
H53	0.8984	0.3381	0.5269	0.047*	
C54	0.9931 (6)	0.4687 (5)	0.5694 (2)	0.0570 (13)	
H54A	1.0390	0.4795	0.6057	0.068*	
H54B	1.0854	0.4732	0.5463	0.068*	
C55	0.8742 (9)	0.5623 (6)	0.5556 (3)	0.0802 (19)	
H55A	0.9302	0.6323	0.5619	0.120*	
H55B	0.7800	0.5575	0.5773	0.120*	
H55C	0.8360	0.5570	0.5187	0.120*	
C56	1.0435 (5)	0.2673 (4)	0.58622 (16)	0.0432 (10)	
H56	1.0662	0.2819	0.6243	0.052*	
C57	0.9728 (7)	0.1508 (4)	0.58050 (19)	0.0544 (12)	
H57A	1.0548	0.0979	0.5941	0.082*	
H57B	0.9442	0.1356	0.5436	0.082*	
H57C	0.8743	0.1450	0.6002	0.082*	
C58	1.2071 (6)	0.2763 (5)	0.5601 (2)	0.0560 (13)	
H58A	1.2544	0.3490	0.5667	0.084*	
H58B	1.1879	0.2652	0.5224	0.084*	
H58C	1.2832	0.2205	0.5744	0.084*	

### Atomic displacement parameters (Å^2^)

**Table:**

	*U*^11^	*U*^22^	*U*^33^	*U*^12^	*U*^13^	*U*^23^
O1	0.0131 (10)	0.0272 (12)	0.0290 (11)	−0.0035 (9)	0.0025 (8)	0.0003 (9)
O2	0.0222 (11)	0.0439 (15)	0.0234 (11)	0.0218 (11)	0.0041 (9)	0.0031 (10)
O3	0.0111 (10)	0.0516 (16)	0.0254 (11)	0.0018 (11)	0.0026 (8)	−0.0117 (11)
O4	0.0097 (9)	0.0317 (13)	0.0318 (11)	−0.0049 (9)	0.0049 (8)	−0.0071 (10)
C1	0.0096 (13)	0.0225 (16)	0.0292 (15)	0.0020 (12)	0.0015 (11)	−0.0021 (13)
C2	0.0234 (15)	0.0126 (14)	0.0305 (16)	0.0035 (12)	0.0007 (12)	−0.0002 (12)
C3	0.0200 (14)	0.0137 (14)	0.0225 (14)	0.0033 (11)	−0.0006 (11)	−0.0019 (11)
C4	0.0135 (13)	0.0091 (13)	0.0283 (15)	0.0058 (10)	−0.0006 (11)	−0.0015 (11)
C5	0.0163 (14)	0.0205 (16)	0.0309 (16)	0.0035 (12)	−0.0031 (12)	0.0014 (12)
C6	0.0143 (14)	0.0124 (13)	0.0328 (16)	0.0034 (11)	0.0036 (11)	−0.0045 (12)
C7	0.0152 (13)	0.0184 (15)	0.0326 (16)	0.0020 (12)	0.0064 (11)	−0.0045 (13)
C8	0.0292 (17)	0.0153 (15)	0.0245 (15)	0.0075 (13)	0.0019 (12)	0.0018 (11)
C9	0.0264 (16)	0.0141 (14)	0.0236 (14)	0.0114 (12)	0.0024 (11)	0.0014 (11)
C10	0.0187 (14)	0.0133 (14)	0.0212 (14)	0.0065 (11)	−0.0010 (11)	−0.0013 (11)
C11	0.0143 (13)	0.0130 (13)	0.0253 (14)	0.0040 (11)	0.0024 (11)	−0.0048 (11)
C12	0.0167 (14)	0.0189 (15)	0.0234 (14)	0.0088 (12)	0.0013 (11)	−0.0026 (12)
C13	0.0144 (13)	0.0166 (15)	0.0269 (14)	0.0000 (12)	0.0019 (11)	−0.0017 (12)
C14	0.0159 (14)	0.0213 (16)	0.0329 (16)	0.0002 (12)	−0.0003 (12)	0.0035 (13)
C15	0.0223 (15)	0.0120 (14)	0.0368 (17)	−0.0002 (12)	0.0083 (13)	−0.0036 (12)
C16	0.0213 (15)	0.0101 (14)	0.0381 (17)	0.0046 (12)	0.0101 (13)	−0.0023 (12)
C17	0.0305 (17)	0.0228 (17)	0.0289 (16)	0.0114 (14)	0.0061 (13)	−0.0008 (13)
C18	0.0268 (16)	0.0286 (18)	0.0288 (16)	0.0105 (14)	0.0067 (13)	0.0002 (14)
C19	0.0158 (14)	0.0237 (16)	0.0295 (16)	0.0000 (12)	0.0033 (12)	−0.0033 (13)
C20	0.0201 (15)	0.0212 (16)	0.0340 (17)	−0.0039 (13)	0.0064 (12)	−0.0009 (13)
C21	0.037 (2)	0.0208 (18)	0.056 (2)	−0.0044 (15)	0.0200 (17)	−0.0009 (16)
C22	0.0230 (16)	0.036 (2)	0.0348 (18)	−0.0072 (15)	0.0071 (13)	−0.0037 (15)
C23	0.032 (2)	0.049 (3)	0.041 (2)	−0.0006 (18)	0.0103 (16)	0.0056 (19)
C24	0.041 (2)	0.047 (2)	0.0302 (18)	−0.0129 (19)	0.0089 (15)	−0.0036 (17)
C25	0.049 (3)	0.052 (3)	0.046 (2)	−0.009 (2)	0.0087 (19)	0.006 (2)
C26	0.104 (5)	0.067 (4)	0.075 (4)	0.018 (4)	0.028 (4)	0.010 (3)
C27	0.031 (2)	0.068 (3)	0.042 (2)	0.004 (2)	0.0075 (16)	0.015 (2)
C28	0.077 (4)	0.059 (4)	0.078 (4)	0.013 (3)	0.011 (3)	−0.007 (3)
C29	0.037 (3)	0.125 (6)	0.067 (3)	0.003 (3)	0.014 (2)	0.041 (4)
C30	0.0089 (13)	0.0367 (19)	0.0254 (15)	0.0018 (13)	0.0035 (11)	−0.0044 (13)
C31	0.0128 (13)	0.0237 (16)	0.0310 (15)	0.0025 (12)	0.0045 (11)	−0.0070 (13)
C32	0.0110 (13)	0.0177 (15)	0.0256 (14)	0.0021 (11)	0.0014 (10)	−0.0009 (12)
C33	0.0083 (12)	0.0187 (14)	0.0216 (13)	−0.0023 (11)	0.0049 (10)	−0.0007 (11)
C34	0.0111 (13)	0.0264 (16)	0.0293 (15)	−0.0025 (12)	0.0028 (11)	−0.0038 (13)
C35	0.0160 (14)	0.0205 (16)	0.0270 (15)	−0.0044 (12)	0.0073 (11)	0.0012 (12)
C36	0.0175 (15)	0.0321 (19)	0.0279 (16)	−0.0020 (13)	0.0092 (12)	0.0006 (13)
C37	0.0165 (14)	0.0139 (14)	0.0297 (15)	−0.0013 (11)	0.0041 (11)	−0.0021 (12)
C38	0.0125 (13)	0.0170 (15)	0.0264 (14)	−0.0039 (11)	0.0037 (11)	0.0011 (11)
C39	0.0099 (12)	0.0136 (13)	0.0230 (14)	−0.0025 (10)	0.0055 (10)	0.0013 (11)
C40	0.0093 (12)	0.0151 (14)	0.0214 (13)	−0.0025 (10)	0.0040 (10)	0.0010 (11)
C41	0.0112 (12)	0.0198 (15)	0.0221 (13)	−0.0047 (11)	0.0046 (10)	0.0007 (11)
C42	0.0139 (13)	0.0195 (15)	0.0206 (13)	−0.0003 (11)	0.0057 (10)	0.0013 (11)
C43	0.0158 (14)	0.0285 (17)	0.0253 (15)	0.0012 (13)	0.0021 (11)	0.0004 (13)
C44	0.0188 (14)	0.0170 (15)	0.0266 (15)	−0.0016 (12)	0.0058 (11)	0.0024 (12)
C45	0.0199 (14)	0.0113 (14)	0.0291 (15)	−0.0043 (11)	0.0085 (12)	0.0006 (11)
C46	0.0214 (15)	0.0267 (18)	0.0295 (16)	−0.0060 (13)	0.0105 (12)	0.0010 (13)
C47	0.0199 (15)	0.0253 (17)	0.0311 (16)	−0.0034 (13)	0.0106 (12)	0.0017 (13)
C48	0.0145 (13)	0.0229 (16)	0.0246 (15)	0.0005 (12)	0.0044 (11)	0.0013 (12)
C49	0.0225 (15)	0.0270 (17)	0.0229 (14)	0.0042 (13)	0.0072 (12)	−0.0017 (13)
C50	0.039 (2)	0.0231 (18)	0.0377 (19)	−0.0024 (15)	0.0105 (15)	−0.0058 (15)
C51	0.0243 (16)	0.0340 (19)	0.0287 (16)	0.0080 (14)	0.0087 (13)	−0.0002 (14)
C52	0.0312 (19)	0.059 (3)	0.0323 (19)	0.0000 (19)	0.0087 (15)	0.0003 (18)
C53	0.040 (2)	0.048 (2)	0.0308 (18)	0.0008 (19)	0.0120 (15)	−0.0002 (17)
C54	0.048 (3)	0.066 (3)	0.059 (3)	0.000 (2)	0.015 (2)	0.011 (3)
C55	0.086 (5)	0.073 (4)	0.082 (4)	0.003 (4)	0.008 (3)	0.028 (4)
C56	0.045 (2)	0.053 (3)	0.0323 (19)	0.005 (2)	0.0090 (16)	−0.0046 (18)
C57	0.064 (3)	0.049 (3)	0.053 (3)	−0.005 (2)	0.020 (2)	−0.002 (2)
C58	0.040 (2)	0.068 (3)	0.062 (3)	0.011 (2)	0.018 (2)	−0.001 (3)

### Geometric parameters (Å, °)

**Table:**

O1—C1	1.436 (4)	C28—H28C	0.9800
O1—H1O	0.8400	C29—H29A	0.9800
O2—C9	1.433 (4)	C29—H29B	0.9800
O2—H2O	0.8400	C29—H29C	0.9800
O3—C30	1.421 (4)	C30—C36	1.515 (5)
O3—H3O	0.8400	C30—C31	1.522 (5)
O4—C38	1.460 (4)	C30—H30	1.0000
O4—H4O	0.8400	C31—C32	1.513 (4)
C1—C7	1.507 (5)	C31—H31A	0.9900
C1—C2	1.523 (4)	C31—H31B	0.9900
C1—H1	1.0000	C32—C37	1.334 (4)
C2—C3	1.511 (4)	C32—C33	1.526 (4)
C2—H2A	0.9900	C33—C34	1.548 (4)
C2—H2B	0.9900	C33—C35	1.550 (4)
C3—C8	1.340 (4)	C33—C40	1.559 (4)
C3—C4	1.531 (4)	C34—H34A	0.9800
C4—C5	1.545 (4)	C34—H34B	0.9800
C4—C6	1.552 (4)	C34—H34C	0.9800
C4—C11	1.558 (4)	C35—C36	1.529 (4)
C5—H5A	0.9800	C35—H35A	0.9900
C5—H5B	0.9800	C35—H35B	0.9900
C5—H5C	0.9800	C36—H36A	0.9900
C6—C7	1.531 (4)	C36—H36B	0.9900
C6—H6A	0.9900	C37—C38	1.491 (4)
C6—H6B	0.9900	C37—H37	0.9500
C7—H7A	0.9900	C38—C39	1.522 (4)
C7—H7B	0.9900	C38—H38	1.0000
C8—C9	1.499 (5)	C39—C41	1.524 (4)
C8—H8	0.9500	C39—C40	1.533 (4)
C9—C10	1.531 (4)	C39—H39	1.0000
C9—H9	1.0000	C40—C45	1.537 (4)
C10—C12	1.517 (4)	C40—H40	1.0000
C10—C11	1.531 (4)	C41—C46	1.531 (4)
C10—H10	1.0000	C41—C42	1.544 (4)
C11—C16	1.542 (4)	C41—H41	1.0000
C11—H11	1.0000	C42—C44	1.533 (4)
C12—C13	1.531 (4)	C42—C43	1.542 (4)
C12—C17	1.531 (4)	C42—C48	1.554 (4)
C12—H12	1.0000	C43—H43A	0.9800
C13—C15	1.540 (4)	C43—H43B	0.9800
C13—C14	1.543 (4)	C43—H43C	0.9800
C13—C19	1.564 (4)	C44—C45	1.536 (4)
C14—H14A	0.9800	C44—H44A	0.9900
C14—H14B	0.9800	C44—H44B	0.9900
C14—H14C	0.9800	C45—H45A	0.9900
C15—C16	1.531 (4)	C45—H45B	0.9900
C15—H15A	0.9900	C46—C47	1.537 (5)
C15—H15B	0.9900	C46—H46A	0.9900
C16—H16A	0.9900	C46—H46B	0.9900
C16—H16B	0.9900	C47—C48	1.552 (5)
C17—C18	1.544 (5)	C47—H47A	0.9900
C17—H17A	0.9900	C47—H47B	0.9900
C17—H17B	0.9900	C48—C49	1.543 (4)
C18—C19	1.553 (5)	C48—H48	1.0000
C18—H18A	0.9900	C49—C50	1.529 (5)
C18—H18B	0.9900	C49—C51	1.534 (4)
C19—C20	1.538 (5)	C49—H49	1.0000
C19—H19	1.0000	C50—H50A	0.9800
C20—C22	1.536 (5)	C50—H50B	0.9800
C20—C21	1.538 (5)	C50—H50C	0.9800
C20—H20	1.0000	C51—C52	1.520 (5)
C21—H21A	0.9800	C51—H51A	0.9900
C21—H21B	0.9800	C51—H51B	0.9900
C21—H21C	0.9800	C52—C53	1.536 (5)
C22—C23	1.521 (5)	C52—H52A	0.9900
C22—H22A	0.9900	C52—H52B	0.9900
C22—H22B	0.9900	C53—C54	1.535 (7)
C23—C24	1.540 (6)	C53—C56	1.538 (6)
C23—H23A	0.9900	C53—H53	1.0000
C23—H23B	0.9900	C54—C55	1.529 (9)
C24—C27	1.527 (7)	C54—H54A	0.9900
C24—C25	1.531 (6)	C54—H54B	0.9900
C24—H24	1.0000	C55—H55A	0.9800
C25—C26	1.481 (9)	C55—H55B	0.9800
C25—H25A	0.9900	C55—H55C	0.9800
C25—H25B	0.9900	C56—C58	1.537 (6)
C26—H26A	0.9800	C56—C57	1.542 (7)
C26—H26B	0.9800	C56—H56	1.0000
C26—H26C	0.9800	C57—H57A	0.9800
C27—C28	1.492 (8)	C57—H57B	0.9800
C27—C29	1.535 (6)	C57—H57C	0.9800
C27—H27	1.0000	C58—H58A	0.9800
C28—H28A	0.9800	C58—H58B	0.9800
C28—H28B	0.9800	C58—H58C	0.9800
			
C1—O1—H1O	109.5	H29A—C29—H29C	109.5
C9—O2—H2O	109.5	H29B—C29—H29C	109.5
C30—O3—H3O	109.5	O3—C30—C36	113.9 (3)
C38—O4—H4O	109.5	O3—C30—C31	111.4 (3)
O1—C1—C7	112.9 (2)	C36—C30—C31	110.6 (3)
O1—C1—C2	107.9 (3)	O3—C30—H30	106.8
C7—C1—C2	110.1 (3)	C36—C30—H30	106.8
O1—C1—H1	108.6	C31—C30—H30	106.8
C7—C1—H1	108.6	C32—C31—C30	111.7 (3)
C2—C1—H1	108.6	C32—C31—H31A	109.3
C3—C2—C1	111.5 (3)	C30—C31—H31A	109.3
C3—C2—H2A	109.3	C32—C31—H31B	109.3
C1—C2—H2A	109.3	C30—C31—H31B	109.3
C3—C2—H2B	109.3	H31A—C31—H31B	107.9
C1—C2—H2B	109.3	C37—C32—C31	119.8 (3)
H2A—C2—H2B	108.0	C37—C32—C33	123.7 (3)
C8—C3—C2	120.4 (3)	C31—C32—C33	116.5 (3)
C8—C3—C4	123.3 (3)	C32—C33—C34	107.9 (2)
C2—C3—C4	116.3 (3)	C32—C33—C35	108.1 (2)
C3—C4—C5	108.1 (2)	C34—C33—C35	109.5 (2)
C3—C4—C6	108.6 (2)	C32—C33—C40	111.2 (2)
C5—C4—C6	109.8 (2)	C34—C33—C40	111.3 (2)
C3—C4—C11	109.4 (2)	C35—C33—C40	108.8 (2)
C5—C4—C11	112.3 (2)	C33—C34—H34A	109.5
C6—C4—C11	108.7 (2)	C33—C34—H34B	109.5
C4—C5—H5A	109.5	H34A—C34—H34B	109.5
C4—C5—H5B	109.5	C33—C34—H34C	109.5
H5A—C5—H5B	109.5	H34A—C34—H34C	109.5
C4—C5—H5C	109.5	H34B—C34—H34C	109.5
H5A—C5—H5C	109.5	C36—C35—C33	114.2 (3)
H5B—C5—H5C	109.5	C36—C35—H35A	108.7
C7—C6—C4	114.2 (3)	C33—C35—H35A	108.7
C7—C6—H6A	108.7	C36—C35—H35B	108.7
C4—C6—H6A	108.7	C33—C35—H35B	108.7
C7—C6—H6B	108.7	H35A—C35—H35B	107.6
C4—C6—H6B	108.7	C30—C36—C35	110.2 (3)
H6A—C6—H6B	107.6	C30—C36—H36A	109.6
C1—C7—C6	111.0 (3)	C35—C36—H36A	109.6
C1—C7—H7A	109.4	C30—C36—H36B	109.6
C6—C7—H7A	109.4	C35—C36—H36B	109.6
C1—C7—H7B	109.4	H36A—C36—H36B	108.1
C6—C7—H7B	109.4	C32—C37—C38	124.1 (3)
H7A—C7—H7B	108.0	C32—C37—H37	117.9
C3—C8—C9	124.9 (3)	C38—C37—H37	117.9
C3—C8—H8	117.5	O4—C38—C37	106.1 (2)
C9—C8—H8	117.5	O4—C38—C39	111.6 (2)
O2—C9—C8	111.1 (2)	C37—C38—C39	112.0 (2)
O2—C9—C10	111.9 (3)	O4—C38—H38	109.0
C8—C9—C10	111.6 (3)	C37—C38—H38	109.0
O2—C9—H9	107.3	C39—C38—H38	109.0
C8—C9—H9	107.3	C38—C39—C41	111.2 (2)
C10—C9—H9	107.3	C38—C39—C40	111.2 (2)
C12—C10—C11	110.6 (2)	C41—C39—C40	110.2 (2)
C12—C10—C9	110.8 (2)	C38—C39—H39	108.0
C11—C10—C9	110.1 (2)	C41—C39—H39	108.0
C12—C10—H10	108.4	C40—C39—H39	108.0
C11—C10—H10	108.4	C39—C40—C45	112.0 (2)
C9—C10—H10	108.4	C39—C40—C33	111.8 (2)
C10—C11—C16	112.2 (2)	C45—C40—C33	113.1 (2)
C10—C11—C4	112.1 (2)	C39—C40—H40	106.5
C16—C11—C4	112.7 (2)	C45—C40—H40	106.5
C10—C11—H11	106.4	C33—C40—H40	106.5
C16—C11—H11	106.4	C39—C41—C46	118.6 (3)
C4—C11—H11	106.4	C39—C41—C42	114.4 (2)
C10—C12—C13	115.0 (2)	C46—C41—C42	104.4 (2)
C10—C12—C17	117.8 (3)	C39—C41—H41	106.2
C13—C12—C17	104.6 (2)	C46—C41—H41	106.2
C10—C12—H12	106.2	C42—C41—H41	106.2
C13—C12—H12	106.2	C44—C42—C43	111.2 (2)
C17—C12—H12	106.2	C44—C42—C41	105.6 (2)
C12—C13—C15	105.7 (2)	C43—C42—C41	112.0 (3)
C12—C13—C14	112.1 (3)	C44—C42—C48	116.9 (3)
C15—C13—C14	111.1 (3)	C43—C42—C48	110.2 (2)
C12—C13—C19	100.7 (2)	C41—C42—C48	100.4 (2)
C15—C13—C19	116.7 (3)	C42—C43—H43A	109.5
C14—C13—C19	110.0 (2)	C42—C43—H43B	109.5
C13—C14—H14A	109.5	H43A—C43—H43B	109.5
C13—C14—H14B	109.5	C42—C43—H43C	109.5
H14A—C14—H14B	109.5	H43A—C43—H43C	109.5
C13—C14—H14C	109.5	H43B—C43—H43C	109.5
H14A—C14—H14C	109.5	C42—C44—C45	111.9 (3)
H14B—C14—H14C	109.5	C42—C44—H44A	109.2
C16—C15—C13	111.0 (3)	C45—C44—H44A	109.2
C16—C15—H15A	109.4	C42—C44—H44B	109.2
C13—C15—H15A	109.4	C45—C44—H44B	109.2
C16—C15—H15B	109.4	H44A—C44—H44B	107.9
C13—C15—H15B	109.4	C44—C45—C40	114.0 (2)
H15A—C15—H15B	108.0	C44—C45—H45A	108.8
C15—C16—C11	114.5 (3)	C40—C45—H45A	108.8
C15—C16—H16A	108.6	C44—C45—H45B	108.8
C11—C16—H16A	108.6	C40—C45—H45B	108.8
C15—C16—H16B	108.6	H45A—C45—H45B	107.7
C11—C16—H16B	108.6	C41—C46—C47	103.5 (3)
H16A—C16—H16B	107.6	C41—C46—H46A	111.1
C12—C17—C18	103.1 (3)	C47—C46—H46A	111.1
C12—C17—H17A	111.1	C41—C46—H46B	111.1
C18—C17—H17A	111.1	C47—C46—H46B	111.1
C12—C17—H17B	111.1	H46A—C46—H46B	109.0
C18—C17—H17B	111.1	C46—C47—C48	107.6 (2)
H17A—C17—H17B	109.1	C46—C47—H47A	110.2
C17—C18—C19	107.6 (3)	C48—C47—H47A	110.2
C17—C18—H18A	110.2	C46—C47—H47B	110.2
C19—C18—H18A	110.2	C48—C47—H47B	110.2
C17—C18—H18B	110.2	H47A—C47—H47B	108.5
C19—C18—H18B	110.2	C49—C48—C47	111.7 (3)
H18A—C18—H18B	108.5	C49—C48—C42	118.5 (3)
C20—C19—C18	111.8 (3)	C47—C48—C42	103.8 (3)
C20—C19—C13	118.7 (3)	C49—C48—H48	107.4
C18—C19—C13	103.7 (3)	C47—C48—H48	107.4
C20—C19—H19	107.4	C42—C48—H48	107.4
C18—C19—H19	107.4	C50—C49—C51	110.3 (3)
C13—C19—H19	107.4	C50—C49—C48	113.1 (3)
C22—C20—C21	109.8 (3)	C51—C49—C48	110.2 (3)
C22—C20—C19	111.0 (3)	C50—C49—H49	107.7
C21—C20—C19	112.8 (3)	C51—C49—H49	107.7
C22—C20—H20	107.7	C48—C49—H49	107.7
C21—C20—H20	107.7	C49—C50—H50A	109.5
C19—C20—H20	107.7	C49—C50—H50B	109.5
C20—C21—H21A	109.5	H50A—C50—H50B	109.5
C20—C21—H21B	109.5	C49—C50—H50C	109.5
H21A—C21—H21B	109.5	H50A—C50—H50C	109.5
C20—C21—H21C	109.5	H50B—C50—H50C	109.5
H21A—C21—H21C	109.5	C52—C51—C49	113.8 (3)
H21B—C21—H21C	109.5	C52—C51—H51A	108.8
C23—C22—C20	114.9 (3)	C49—C51—H51A	108.8
C23—C22—H22A	108.6	C52—C51—H51B	108.8
C20—C22—H22A	108.6	C49—C51—H51B	108.8
C23—C22—H22B	108.6	H51A—C51—H51B	107.7
C20—C22—H22B	108.6	C51—C52—C53	114.9 (3)
H22A—C22—H22B	107.5	C51—C52—H52A	108.5
C22—C23—C24	116.7 (3)	C53—C52—H52A	108.5
C22—C23—H23A	108.1	C51—C52—H52B	108.5
C24—C23—H23A	108.1	C53—C52—H52B	108.5
C22—C23—H23B	108.1	H52A—C52—H52B	107.5
C24—C23—H23B	108.1	C54—C53—C52	112.6 (4)
H23A—C23—H23B	107.3	C54—C53—C56	110.9 (4)
C27—C24—C25	113.3 (3)	C52—C53—C56	112.7 (4)
C27—C24—C23	112.9 (4)	C54—C53—H53	106.7
C25—C24—C23	110.5 (4)	C52—C53—H53	106.7
C27—C24—H24	106.6	C56—C53—H53	106.7
C25—C24—H24	106.6	C55—C54—C53	115.5 (5)
C23—C24—H24	106.6	C55—C54—H54A	108.4
C26—C25—C24	114.3 (4)	C53—C54—H54A	108.4
C26—C25—H25A	108.7	C55—C54—H54B	108.4
C24—C25—H25A	108.7	C53—C54—H54B	108.4
C26—C25—H25B	108.7	H54A—C54—H54B	107.5
C24—C25—H25B	108.7	C54—C55—H55A	109.5
H25A—C25—H25B	107.6	C54—C55—H55B	109.5
C25—C26—H26A	109.5	H55A—C55—H55B	109.5
C25—C26—H26B	109.5	C54—C55—H55C	109.5
H26A—C26—H26B	109.5	H55A—C55—H55C	109.5
C25—C26—H26C	109.5	H55B—C55—H55C	109.5
H26A—C26—H26C	109.5	C58—C56—C53	111.5 (4)
H26B—C26—H26C	109.5	C58—C56—C57	110.8 (4)
C28—C27—C24	113.8 (4)	C53—C56—C57	111.4 (4)
C28—C27—C29	111.1 (5)	C58—C56—H56	107.6
C24—C27—C29	110.8 (5)	C53—C56—H56	107.6
C28—C27—H27	106.9	C57—C56—H56	107.6
C24—C27—H27	106.9	C56—C57—H57A	109.5
C29—C27—H27	106.9	C56—C57—H57B	109.5
C27—C28—H28A	109.5	H57A—C57—H57B	109.5
C27—C28—H28B	109.5	C56—C57—H57C	109.5
H28A—C28—H28B	109.5	H57A—C57—H57C	109.5
C27—C28—H28C	109.5	H57B—C57—H57C	109.5
H28A—C28—H28C	109.5	C56—C58—H58A	109.5
H28B—C28—H28C	109.5	C56—C58—H58B	109.5
C27—C29—H29A	109.5	H58A—C58—H58B	109.5
C27—C29—H29B	109.5	C56—C58—H58C	109.5
H29A—C29—H29B	109.5	H58A—C58—H58C	109.5
C27—C29—H29C	109.5	H58B—C58—H58C	109.5
			
O1—C1—C2—C3	−179.6 (2)	O3—C30—C31—C32	177.6 (2)
C7—C1—C2—C3	−56.0 (3)	C36—C30—C31—C32	−54.6 (3)
C1—C2—C3—C8	−127.2 (3)	C30—C31—C32—C37	−125.8 (3)
C1—C2—C3—C4	52.8 (3)	C30—C31—C32—C33	52.0 (3)
C8—C3—C4—C5	−108.6 (3)	C37—C32—C33—C34	−112.4 (3)
C2—C3—C4—C5	71.4 (3)	C31—C32—C33—C34	70.0 (3)
C8—C3—C4—C6	132.3 (3)	C37—C32—C33—C35	129.4 (3)
C2—C3—C4—C6	−47.6 (3)	C31—C32—C33—C35	−48.3 (3)
C8—C3—C4—C11	13.9 (4)	C37—C32—C33—C40	10.0 (4)
C2—C3—C4—C11	−166.0 (2)	C31—C32—C33—C40	−167.7 (2)
C3—C4—C6—C7	48.7 (3)	C32—C33—C35—C36	50.8 (3)
C5—C4—C6—C7	−69.2 (3)	C34—C33—C35—C36	−66.4 (3)
C11—C4—C6—C7	167.6 (2)	C40—C33—C35—C36	171.7 (2)
O1—C1—C7—C6	178.6 (2)	O3—C30—C36—C35	−176.2 (3)
C2—C1—C7—C6	57.9 (3)	C31—C30—C36—C35	57.4 (3)
C4—C6—C7—C1	−56.2 (3)	C33—C35—C36—C30	−57.4 (3)
C2—C3—C8—C9	−178.1 (3)	C31—C32—C37—C38	179.3 (3)
C4—C3—C8—C9	2.0 (5)	C33—C32—C37—C38	1.7 (5)
C3—C8—C9—O2	−111.9 (3)	C32—C37—C38—O4	−105.2 (3)
C3—C8—C9—C10	13.7 (4)	C32—C37—C38—C39	16.8 (4)
O2—C9—C10—C12	−42.1 (3)	O4—C38—C39—C41	−51.0 (3)
C8—C9—C10—C12	−167.3 (3)	C37—C38—C39—C41	−169.8 (2)
O2—C9—C10—C11	80.6 (3)	O4—C38—C39—C40	72.2 (3)
C8—C9—C10—C11	−44.6 (3)	C37—C38—C39—C40	−46.5 (3)
C12—C10—C11—C16	−46.3 (3)	C38—C39—C40—C45	−172.5 (2)
C9—C10—C11—C16	−169.1 (3)	C41—C39—C40—C45	−48.6 (3)
C12—C10—C11—C4	−174.3 (2)	C38—C39—C40—C33	59.4 (3)
C9—C10—C11—C4	62.9 (3)	C41—C39—C40—C33	−176.7 (2)
C3—C4—C11—C10	−45.6 (3)	C32—C33—C40—C39	−39.8 (3)
C5—C4—C11—C10	74.3 (3)	C34—C33—C40—C39	80.6 (3)
C6—C4—C11—C10	−164.0 (2)	C35—C33—C40—C39	−158.7 (2)
C3—C4—C11—C16	−173.3 (2)	C32—C33—C40—C45	−167.3 (2)
C5—C4—C11—C16	−53.4 (3)	C34—C33—C40—C45	−47.0 (3)
C6—C4—C11—C16	68.3 (3)	C35—C33—C40—C45	73.8 (3)
C11—C10—C12—C13	56.7 (3)	C38—C39—C41—C46	−54.3 (3)
C9—C10—C12—C13	179.1 (2)	C40—C39—C41—C46	−178.1 (3)
C11—C10—C12—C17	−179.2 (3)	C38—C39—C41—C42	−178.2 (2)
C9—C10—C12—C17	−56.9 (3)	C40—C39—C41—C42	58.0 (3)
C10—C12—C13—C15	−61.8 (3)	C39—C41—C42—C44	−61.4 (3)
C17—C12—C13—C15	167.4 (3)	C46—C41—C42—C44	167.4 (2)
C10—C12—C13—C14	59.3 (3)	C39—C41—C42—C43	59.7 (3)
C17—C12—C13—C14	−71.4 (3)	C46—C41—C42—C43	−71.5 (3)
C10—C12—C13—C19	176.3 (2)	C39—C41—C42—C48	176.6 (2)
C17—C12—C13—C19	45.6 (3)	C46—C41—C42—C48	45.4 (3)
C12—C13—C15—C16	58.3 (3)	C43—C42—C44—C45	−64.1 (3)
C14—C13—C15—C16	−63.5 (3)	C41—C42—C44—C45	57.6 (3)
C19—C13—C15—C16	169.3 (3)	C48—C42—C44—C45	168.2 (3)
C13—C15—C16—C11	−54.6 (4)	C42—C44—C45—C40	−54.5 (3)
C10—C11—C16—C15	47.4 (4)	C39—C40—C45—C44	48.5 (3)
C4—C11—C16—C15	175.1 (3)	C33—C40—C45—C44	176.0 (2)
C10—C12—C17—C18	−165.3 (3)	C39—C41—C46—C47	−163.5 (3)
C13—C12—C17—C18	−36.2 (3)	C42—C41—C46—C47	−34.9 (3)
C12—C17—C18—C19	12.4 (3)	C41—C46—C47—C48	10.5 (3)
C17—C18—C19—C20	144.2 (3)	C46—C47—C48—C49	146.1 (3)
C17—C18—C19—C13	15.1 (3)	C46—C47—C48—C42	17.3 (3)
C12—C13—C19—C20	−161.2 (3)	C44—C42—C48—C49	84.2 (3)
C15—C13—C19—C20	85.0 (4)	C43—C42—C48—C49	−44.0 (4)
C14—C13—C19—C20	−42.7 (4)	C41—C42—C48—C49	−162.2 (3)
C12—C13—C19—C18	−36.5 (3)	C44—C42—C48—C47	−151.3 (3)
C15—C13—C19—C18	−150.3 (3)	C43—C42—C48—C47	80.5 (3)
C14—C13—C19—C18	82.0 (3)	C41—C42—C48—C47	−37.7 (3)
C18—C19—C20—C22	59.9 (4)	C47—C48—C49—C50	−179.1 (3)
C13—C19—C20—C22	−179.5 (3)	C42—C48—C49—C50	−58.5 (4)
C18—C19—C20—C21	−176.4 (3)	C47—C48—C49—C51	56.9 (3)
C13—C19—C20—C21	−55.7 (4)	C42—C48—C49—C51	177.5 (3)
C21—C20—C22—C23	70.0 (4)	C50—C49—C51—C52	80.5 (4)
C19—C20—C22—C23	−164.7 (3)	C48—C49—C51—C52	−153.8 (3)
C20—C22—C23—C24	−173.5 (3)	C49—C51—C52—C53	170.2 (4)
C22—C23—C24—C27	−58.9 (5)	C51—C52—C53—C54	−63.0 (5)
C22—C23—C24—C25	173.2 (4)	C51—C52—C53—C56	63.3 (5)
C27—C24—C25—C26	162.0 (5)	C52—C53—C54—C55	−45.6 (6)
C23—C24—C25—C26	−70.2 (6)	C56—C53—C54—C55	−172.9 (4)
C25—C24—C27—C28	63.1 (5)	C54—C53—C56—C58	−54.0 (5)
C23—C24—C27—C28	−63.4 (5)	C52—C53—C56—C58	178.7 (4)
C25—C24—C27—C29	−62.9 (5)	C54—C53—C56—C57	−178.4 (4)
C23—C24—C27—C29	170.6 (4)	C52—C53—C56—C57	54.3 (5)

### Hydrogen-bond geometry (Å, °)

**Table:**

*D*—H···*A*	*D*—H	H···*A*	*D*···*A*	*D*—H···*A*
O1—H1O···O4	0.84	1.92	2.762 (3)	178
O2—H2O···O3	0.84	1.87	2.686 (3)	165
O3—H3O···O1^i^	0.84	1.86	2.694 (3)	170

Symmetry codes: (i) *x*−1, *y*, *z*.
